# Measured Submersion Times in Underwater Hockey Are Inconsistent With Its Classification as an Extreme Apneic Sport

**DOI:** 10.7759/cureus.41816

**Published:** 2023-07-13

**Authors:** Lucky Meisenheimer, John Meisenheimer, Jake A Meisenheimer

**Affiliations:** 1 Internal Medicine, Division of Dermatology, Orlando Regional Medical Center, Orlando, USA; 2 Internal Medicine, University of South Florida Morsani College of Medicine, Tampa, USA; 3 Programming, Hilton Software, Coral Springs, Florida, USA

**Keywords:** octopush, shallow water blackout, aquatic sports medicine, safety, apneic sports, breath control, hypoxic blackout, submersion times, underwater hockey

## Abstract

Underwater hockey (UWH) is a sport played at the bottom of a pool without the use of breathing devices such as scuba equipment. It has been classified as an extreme apneic sport based on perceptions of prolonged underwater submersion times during play. This study measured 2000 submersion times during UWH games and compared the average measured submersion times to estimates by UWH players and aquatics directors. The average measured submersion time was 11.0 seconds (SD:3.7) with a range of 4 to 27 seconds, but aquatics directors' estimates were over 100 percent longer (22.7 seconds). While observed active drop times typically lasted for 12.1 seconds (SD: 3.7), observed drop times with no puck lasted on average 9.3 seconds (SD:3.0). When compared to director and player estimates, actual/observed drop times were significantly (p<0.05) lower for overall drop times, active drop times, and drop times without a puck. The average submersion times measured in this study more closely resembled competitive swimming, a breathing-controlled sport, and contradicted lay press reports of routine submersion for one to three minutes, which implies a risk for a hypoxic blackout. The results of this study may mitigate safety concerns about UWH as a high-risk sport for a hypoxic blackout.

## Introduction

Underwater hockey (UWH) is played worldwide in over 40 countries, recognized by the International Olympic Committee, governed by the World Underwater Federation (also known as Confédération Mondiale des Activités Subaquatiques or CMAS), and has held national and world championships for four decades [1,2]. The sport is played in a flat-bottomed pool with a water depth typically between six and 12 feet. Participants play the game underwater, wearing fins, a snorkel, and a mask. They use a small stick (under 12 inches long) held in a thickly gloved hand to push a three-pound plastic-encased puck along the bottom of the pool into the opposing goal. Teams consist of six active players and four reserve players. Each member can freely substitute with a teammate at any time during play. It is a limited-contact sport in which players cannot push, shove, or impede competitors’ movements [3]. Teamwork is essential to the game. Players make quick drops underwater to play their position then return to the surface. If a player feels winded at the surface, they are expected to sub-out [4].

Perceived long apneic times during underwater submersion in UWH have led it to be categorized as an apneic aquatic sport, along with free diving, static apnea, spearfishing, and apneic underwater swimming, where breath-hold times can be three minutes or more, and which have resulted in deaths attributed to dangerous underwater breath-holding behaviors [5,6]. However, UWH more closely resembles competitive swimming, a breath-controlled, rather than apneic, aquatic sport.

Swimmers hold their breath in and out of turns, representing more than one-third of race time in all events 200 yards or longer [7]. It is not uncommon for elite competitive swimmers to remain underwater for 10 to 15 meters during turns [8]. Olympic swimming sportscaster and triple gold medalist Rowdy Gaines explains that the breath-hold in freestyle swimming commences during the approach to the wall, and swimmers racing at maximum efforts can have repeated 6 to 8-second breath-holds during turns (Rowdy Gaines personal communication July 2021).

Lay press and UWH players commonly report remaining submerged for 2 to 3 minutes before surfacing [9,10]. Physiological studies of UWH players have also used 45-second out-of-water apneic intervals to simulate what were assumed to be conditions experienced in UWH [11]. Such assumptions about prolonged apneic conditions have raised safety concerns about the risk of hypoxic blackout (also known as shallow water blackout) drowning in UWH. Some aquatic facilities prohibit breath-holding activities,” therefore, perceptions about actual apneic times during UWH can affect aquatic program directors’ decisions about making facilities available for the sport [12].

A hypoxic blackout is a concern for all aquatic activities, and studies have examined the circumstances surrounding these events [13,14]. A hypoxic blackout is a loss of consciousness resulting from cerebral hypoxia secondary to deliberate hyperventilation before a dive in order to lower carbon dioxide levels and block the urge to breathe [15-17]. In many cases of hypoxic blackouts, there has been hyperventilation before the breath-hold [18]. Although Davis and Graves demonstrated increased tolerance to CO_2_ levels in trained UWH players, according to the past president of the Underwater Society of America, there have been no reported hypoxic blackouts or deaths in UWH tournaments or organized practices worldwide (C. Rose, personal communication, July 12, 2021) [19]. If, as suggested by players and the lay press, significantly prolonged apneic episodes were commonly experienced during UWH, then hypoxic blackout deaths would be expected to have been documented in this sport. Nevertheless, UWH appears on lists of sports at-risk for hypoxic blackout, based on the assumption that players are repeatedly underwater for long apneic periods and underwater swimming blackouts have occurred in as little as 40 seconds [5,20].

This study seeks to determine whether safety concerns for UWH are justified or if they are based on its misperception as an extreme apneic sport. Breath control is a requirement for all aquatic sports, and in UWH is measured by submerged time underwater. This study is the first to document the actual amount of time UWH players spend underwater with each dive during competition and compare these to players’ and aquatics directors’ perceptions of these times.

## Materials and methods

Two thousand UWH “drops” (the time between the submersion of the player’s head/snorkel below the surface of the water during play to the moment of resurfacing the head/snorkel above the surface) were analyzed using underwater videography. A variety of ability levels were observed, from club scrimmages to national championships and world championship play. However, not all drop times in each game could be measured. Due to limitations on the underwater camera’s field of view and depth of field, and other players or water turbidity obscuring subjects, the study only collected drop times when the reviewer could determine the player’s submersion and resurfacing points. In addition, the study did not include partial drops (a partial drop is when the head/snorkel submerges temporarily, but the athlete does not enter the field of play).

The data collected from the drops included the player’s sex and length of drop time in seconds rounded to the nearest second. Whether there was contact with the puck by the player during the drop was also recorded. Average drop times were calculated for all players in the study, for all men and all women in the study, for when there was contact with the puck by men or women players, for when there was no contact with the puck by men or women, and for recreational club play versus elite world championship play.

Competitive players completed online surveys on their perceptions of how long they believed players remained submerged. They were asked how long they believed their average breath-hold times were for each drop during gameplay, what they believed their average drop time (breath-hold time) was when they actively played the puck, and what they believed their average breath-hold time was when they dropped but had no contact with the puck. Demographic data were also collected on the player’s sex, years of playing underwater hockey, and level of play: “Elite” (played on a national elite team in any world championships), “A player” (played on a national age group team or national masters team in worlds and/or on A division team at your country’s National Championships), “B player” (been in several tournaments and/or has played a B division team at your country’s national championships), “C player” (played in some tournaments, knew the rules but would not fit well on an A or B team at Nationals), or “Novice” (less than a year of experience playing underwater hockey or no or minimal tournament play, mostly recreational play).

Aquatics program directors completed a separate online survey asking them to rate their familiarity with UWH: 1) unfamiliar with the sport before receiving the survey to having played UWH, 2) knew of UWH or read about it, but never saw it played, 3) watched videos of UWH or watched it live, 4) played UWH in practice or competition. They were told that the sport requires players to repeatedly swim to the bottom of the pool, where play with the puck occurs, and then back to the surface of the water to recover. They were then asked how long they perceived players to be underwater, on average, each time they swam down until they resurfaced during scrimmages or games. They were not asked to differentiate between drops that involved contact with the puck or not. The results of the surveys were tabulated and compared to the measured data. For determining whether differences existed between various groups the null hypothesis of no difference between groups was tested using analysis of variance (ANOVA) with an alpha level of 0.05.

## Results

A total of 2000 drops were timed and recorded from observation of 10 matches captured on underwater video, including regional, national, and world championship games. Observers recorded actual drop times from random individual players. One hundred fifty (n=150) competitive hockey players and forty-four (n=44) aquatics directors completed the online survey. The majority of observed drops (59.7%, n=1194) occurred during active play. On average, players were observed to be submerged for an average of 11.0 seconds (SD:3.7) overall. The longest drop time recorded in the study was 27.0 seconds by an elite male player during a world championship game. The shortest drop time was 4 seconds. While observed active drop times typically lasted for 12.1 seconds (SD: 3.7), observed drop times with no puck lasted on average 9.3 seconds (SD:3.0). A statistically significant association was observed between actual drop time and whether the player was in active play.

Directors and hockey players reported higher estimated drop times compared to actual, observed drop times. When comparing director and player estimates, actual/observed drop times were significantly (p<0.05) lower for overall drop times, active drop times, and drop times without a puck (Table 1), with directors reporting the least accurate estimates.

**Table 1:** Comparison Between Observed Drop Times and Estimated Drop Times by Aquatic Directors and Players Videography analyzed in the studies included films of UWH club games 2015 & 2018 and U.S. National Championships 2019 hosted at the YMCA Aquatic Center in Orlando, Florida, and World Championships 2018 Centre Aquatique Desjardins, Quebec

Drop Time	Observed	Director	Player	p
Drop Time (Overall)				
Mean (SD)	11.0 secs (3.7)	22.7 secs (19.8)	15.2 secs (8.4)	<0.001
Median (Interquartile Range)	10 secs (8-13)	16.5 secs (10-30)	12 secs (10-20)	0.02
Drop Time (Active)				
Mean (SD)	12.1 secs (3.7)	-	14.7 secs (9.0)	<0.001
Median (Interquartile Range)	12 secs (9-14)	-	12 secs (9-17)	0.02
Drop Time (No Puck)				
Mean (SD)	9.3 secs (3.0)	-	13.8 secs (10.8)	<0.001
Median (Interquartile Range)	9 secs (7-11)	-	10 secs (6-20)	<0.001

Aquatic directors displayed a low level of familiarity with underwater hockey, with only 15.9% of directors having ever played underwater hockey (Table 2). Median estimated drop times appeared to vary significantly (p=0.04) depending on the level of familiarity, with those not familiar and those who played underwater hockey having the highest estimates.

**Table 2:** Aquatic Director Familiarity With Underwater Hockey

Level of Familiarity	N (%)	Mean (SD)	Median (IQR)
Level of Familiarity (seconds)			p=0.04
Not Familiar	10 (22.7%)	22.5 (16.2)	20 (10-30)
Heard of It, Never Played It	14 (31.8%)	15.8 (6.7)	15 (10-15)
Watched Underwater Hockey	13 (29.6%)	30.0 (31.9)	18 (10-35)
Played Underwater Hockey	7 (15.9%)	23.3 (8.1)	25 (20-30)

The majority of UWH players that completed the survey were male (77.6%, n=116). Only 6% (n=9) players reported being a Novice while 22.7% (n=34) reported being an Elite player (Table 3). Most players had 15 years of experience or more (34.0%, n=51), followed by five to nine years (27.3%, n=41), <5 years (22.0%, n=33), and 10-14 years (16.7%, n=25). Player characteristics, including gender, player level, and years of experience, did not appear associated with estimated drop times (p>0.05).

**Table 3:** Hockey Player Characteristics and Estimated Drop Times

Characteristic	N (%)	Overall Drop Time (Secs)		Active Drop Time (Secs)		No Puck Drop Time (Secs)	
		Mean (SD)	Median (IQR)	Mean (SD)	Median (IQR)	Mean (SD)	Median (IQR)
Sex			p=0.21		p=0.50		p=0.70
Male	116 (77.3%)	15.0 (8.5)	12 (10-20)	14.2 (8.5)	12 (9-15)	13.5 (10.5)	10 (6-20)
Female	34 (22.7%)	15.8 (8.1)	15 (10-20)	16.6 (10.6)	15 (10-20)	14.9 (12.0)	10 (8-20)
Player Level			p=0.70		p=0.90		p=0.22
Elite	34 (22.7%)	13.6 (6.9)	10 (9-20)	14.3 (8.6)	12 (8-17)	9.9 (6.2)	7 (5-15)
A-Level	48 (32.0%)	15.7 (8.7)	12.5 (10-20)	14.9 (10.2)	12.5 (10-15)	15.1 (12.4)	10 (7-20)
B-Level	40 (26.7%)	16.0 (7.2)	15 (10-21)	15.6 (8.9)	14 (10-18.5)	16.5 (11.6)	14 (8-20)
C-Level	19 (12.7%)	14.2 (7.8)	12 (7-20)	13.9 (8.1)	10 (7-23)	14.4 (11.7)	10 (5-20)
Novice	9 (6.0%)	12.7 (6.9)	12 (8-15)	12.7 (6.9)	12 (8-15)	8.4 (5.1)	9 (5-10)
Experience (Years)			p=0.30		p=0.73		p=0.12
<5 Years	33 (22.0%)	14.6 (9.3)	10 (10-20)	13.5 (7.3)	12 (8-15)	13.8 (10.4)	10 (7-20)
5-9 Years	41 (27.3%)	16.3 (8.9)	15 (10-20)	16.7 (12.5)	15 (9-20)	16.3 (13.2)	12 (7-20)
10-14 Years	25 (16.7%)	13.4 (7.9)	10 (8-15)	14.7 (8.5)	13 (10-17)	10.4 (7.7)	9 (5-11)
>=15 Years	51 (34.0%)	15.5 (7.5)	14 (10-20)	14.0 (6.6)	12 (10-16)	13.5 (10.1)	10 (6-20)

## Discussion

The data demonstrate an extreme divide between the perceived submersion times of UWH players and actual measured underwater times. Surprisingly, elite players’ predictions of drop times were not statistically different from other ability levels. Aquatic directors had the worst estimates of how long UWH players stayed submerged, missing the actual times by over 100%. Even experienced players' perceptions were off by 38%.

The recorded periods of breath control of UWH players (9 to 12 seconds) approach the breath-control patterns experienced by competitive swimmers (6 to 8 seconds) during swimming turns. Extremely long apneic periods are not seen in either sport. Out of the 2000 drops recorded in this study, the longest was 27 seconds by an elite world-class player, a fraction of the time the lay press promotes as maximal submerged times for this sport.

Although breath-control comparisons between swimming and UWH are reasonable based on underwater time parameters, breathing patterns can differ considerably based on the nature of each competitive activity. In competitive swimming, the athlete is performing at maximal effort throughout the race. There is no resting phase before entering into turns when most breath-holding takes place. On the other hand, UWH has short rest phases on the surface and minimal physical effort during descent and ascent. Additionally, UWH players relax underwater unless actively involved in the play. Furthermore, there are no substitutions in a swim race. Based on these differences it would appear breath control is on a similar level in both sports.

With regard to drop times during active play specifically, it is not surprising that the average UWH submersion time was 12.1 seconds when the player had contact with the puck versus 9.3 seconds when in the field of play without interaction. There is more motivation to stay down longer when a player is actively contacting the puck. However, the fact that not all drop times during games could be recorded is a limitation of this study. Factors involved in this limitation included the narrow field of view of the videography, obstruction of view by other players, and in some cases, excessive bubbles and turbidity in the water. Measurements of all drop times in a competition might have understandably altered the average, given the possibility that a longer drop time than the recorded maximum of 27 seconds could have occurred and remained undocumented. Further studies on drop times would be beneficial to further assess apneic periods during UWH. Future studies on drop times should look at how age, sex, fitness level, duration of play, and player rankings may affect individuals' submersion times.

The submersion times observed in this study contrast past physiological studies of UWH players that have used 45-second apneic periods with exercise to simulate UWH play, about 400% longer than the drop times measured here. Future physiological simulations of UWH activities might assess breath-control periods of 9 to 12 seconds in order to replicate the average breathing patterns of players more realistically.

Concerns regarding risks of hypoxic blackout due to extreme apneic periods in UWH do not seem to be substantiated by the results of this study. The shorter apneic periods observed in this study may explain why there have not been any recorded hypoxic blackout deaths during organized UWH practices and competitions in over 60 years of the sport. Hypoxic blackouts remain a concern for aquatic sports participants who practice dangerous underwater breathing behaviors, especially hyperventilation before long submersions. However, this type of extreme apneic breath-holding does not appear consistent with UWH.

## Conclusions

In the sport of UWH, safety concerns due to misperceived long apneic times appear misguided. This study documents that UWH players' and aquatic program directors’ perceptions of players’ time underwater are not consistent with measured submersion times. The average measured submersion time of 11.0 seconds refutes lay press and players’ perceptions of repeated one to three-minute submerged times during games. Furthermore, average perceived submersion times by players were 38% longer than measured times, and aquatics directors’ predicted submerged times were 106% longer than recorded measured times.

This study suggests that UWH should be categorized as a breath-control sport, like competitive swimming, rather than an extreme apneic sport. The fact that hypoxic blackout deaths have not been reported in relation to UWH may be because actual submersion times are much shorter than perceived by players and aquatic directors. This new data should help aquatics directors make better-informed safety decisions on the use of their facilities for UWH tournaments and teams. Nevertheless, as in all aquatic sports, risks of dangerous underwater breathing behaviors exist, especially hyperventilation before long submersions. Hyperventilation before long breath holds should continue to be discouraged in all aquatic sports and training sessions, including UWH.
